# Stricture of the Female Urethra

**Published:** 1887-07

**Authors:** 


STRICTURE OF THE FEMALE URETHRA.—*Dr. T. A Ashby* reported a case of stricture of the urethra in a mulatto woman. The urethra was almost entirely closed and urine could only be voided drop by drop. One stricture was located at the external meatus and another about midway of the canal. The urethra would only admit of the passage of a very small probe. Dilatation was accomplished until a No. 12 catheter was passed. The cause of the stricture could not be altogether satisfactorily accounted for. There was no history of gonorrhœa or of syphilis. The mucous and submucous tissues surrounding the urethra were much thickened and indurated by a deposit of fibrous tissue without any discoverable local cause. Stricture of the female urethra is an exceeding uncommon condition and the literature of the subject is not extensive. In a paper recently ead before the Obstetrical Society of London, Dr. Herman had only been able to collect the reports of twenty-three cases, six of which had occurred under his own observation.

The causes of stricture of the female urethra may be the same as in the male. In young and middle aged subjects, gonorrhœa, the cicatrization of chancroids, injury from childbearing, are the chief factors at work in the production of the condition. In old women, induration and thickening of the urethra-vaginal cellular tissue may cause a stenosis of the canal. The case reported by Dr. Ashby seemed to belong to the latter class, although the age of this patient was only forty years.—*Maryland Medical Journal.*

